# Supplementary material for: Collaborative intelligence in AI: Evaluating the performance of a council of AIs on the USMLE
Source: PLOS Digit Health. 2025 Oct 9;4(10):e0000787. doi: 10.1371/journal.pdig.0000787 (PMC12510544; doi:10.1371/journal.pdig.0000787)
Supplement: S2 File — (ZIP) [file pdig.0000787.s002.zip › S2 Data Raw data files containing unprocessed question inputs and LLM outputs including transcripts of deliberations/STEP3_Question-87-1_Type-MC-NJ.docx]

**Facilitator** **[Round 1]:** A 40-year-old woman, gravida 2 para 2, comes to the office because of bulging veins in her legs that have slowly become more visible since she first noticed them 2 years ago. She says they seem to worsen when she stands for long periods of time, but she reports no associated pain. She is concerned that this problem may be life-threatening. Medical history is otherwise unremarkable and she takes no medications. Both of her pregnancies resulted in uncomplicated vaginal deliveries at 40 weeks' gestation. BMI is 23 kg/m². Vital signs are normal. Physical examination discloses compressible veins in both lower extremities below the knees that are worse on the left. The remainder of the examination discloses no abnormalities. Which of the following is the most appropriate next step? (A) Compression stockings (B) Surgical ligation of the largest veins (C) Venography (D) Venous duplex ultrasonography (E) Warfarin therapy

**AI_A:** 1) RESTATING THE QUESTION: The question is referring to a 40-year-old woman who is experiencing a visible increase in the presence of leg veins over the course of two years. These veins become more conspicuously inflated when she stands for long stretches of time. Despite this, the woman reports no pain associated with this condition. Given her otherwise healthy medical history, she is concerned that this seemingly isolated issue may pose a serious risk to her health. The physical examination confirms the presence of these veins, particularly towards the lower part of her body, and also notes that the condition appears to be worse in the left leg. The rest of her examination did not reveal any other concerns. Given these circumstances, the question is asking which would be the most logical next step in medical intervention. 2) PLANNING: To address this situation, we assume this woman's condition to be chronic venous insufficiency, a common issue as a result of valve incompetence in the venous system often exacerbated by standing for prolonged periods. We could also consider other conditions such as deep vein thrombosis, but in this case, the likelihood is low due to lack of pain and the slow gradual visible change over 2 years. As such, the focus should be diagnosing the extent and the severity of chronic venous insufficiency. 3) CLINICAL REASONING: In cases of chronic venous insufficiency, initial diagnostic steps often involve non-invasive techniques to visualize the veins. While compressive therapies are indeed commonly suggested for symptom management of this condition, it is necessary to understand the extent of the problem prior to committing to a treatment plan. Surgical intervention and the use of anticoagulant medication such as Warfarin would ideally be the last resort or when there's a significant risk of severe complications already identified, which is not the case here. 4) CONCLUDING: Hence, in this patient's case, the clinical management should begin with diagnostic investigations into the presence of venous incompetences or other venous disorders. 5) RESPONSE: Therefore, the most appropriate next step would be to conduct a (D) Venous duplex ultrasonography, which is a noninvasive evaluation that provides both anatomic and functional information about the veins and is often a first-line investigation in patients with chronic venous insufficiency.

**AI_B:** (1) RESTATING THE QUESTION: We are given the case of a generally healthy 40-year-old woman who has been noticing increasingly visible bulging veins in her legs over the last two years, worsening with long periods of standing. Despite her apprehension, she doesn't have any associated pain. Her only known medical history involves two uncomplicated pregnancies and deliveries, and our physical exam reveals compressible veins below the knees, more pronounced on her left side. Here, the concern relates to venous insufficiency, which refers to the inability of the veins to maintain adequate return of blood from the peripheral tissues to the heart. The question is asking for the best next step in managing her case. (2) PLANNING: The potential assumptions could include that she has (i) uncomplicated varicose veins, (ii) varicose veins with a deep vein thrombosis (given the asymmetry), or (iii) a different condition altogether. Alternate assumptions could involve questioning the above. In clinically prioritizing, we should consider the impact on her quality of life, the potential consequences of an unmanaged condition, and the course of action that could address the most pressing concern with the minimum risk to the patient. (3) CLINICAL REASONING: While her physical exam is consistent with varicose veins, a type of chronic venous insufficiency often caused by damaged or weakened valves in the veins, it's important to confirm whether she has simple uncomplicated varicosis, or if there's an underlying deep venous pathology (as reflected by the asymmetry). Her condition seems stable without any associated symptoms like pain, edema, or skin changes, which suggests uncomplicated varicose veins. However, the cause of the varicosities (whether primary or secondary) still needs to be determined before commencing definitive treatment. (4) CONCLUDING: Consequently, a step-by-step investigation is warranted to confirm our suspicion and understand the underlying cause. An imaging technique, such as venous duplex ultrasonography, would serve this purpose. It allows visualization of the venous system and assesses the venous valve competency effectively while being noninvasive and without radiation exposure. This will provide a clearer picture of our assumptions and help manage the patient more effectively. (5) RESPONSE: Therefore, the most appropriate next step in this case would be to order a Venous duplex ultrasonography (Choice D) to better gauge the condition of her veins and guide subsequent management.

**AI_C:** RESTATING THE QUESTION: The situation revolves around a 40-year-old woman presenting with bulging veins in her legs that become more pronounced when she stands for long periods. She has given birth twice without any complications and is otherwise healthy, aside from the noticeable veins that are more severe on her left side. The question here is, given her condition, what would be the most suitable next step in terms of medical management? PLANNING: We'll need to evaluate the possibility of different diseases, such as varicose veins (which is suggested by the presence of "bulging veins" which worsen on standing), deep vein thrombosis (where the clot formed in deep veins can cause swelling, but usually associated with pain and tenderness), etc. Since the patient's symptoms are typical for varicose veins, and she has no other symptoms suggesting a risk of deep vein thrombosis or other complications, most likely the diagnosis is varicose veins. CLINICAL REASONING: Varicose veins are dilated, tortuous veins that form because of defective valves within the veins, allowing blood to pool, resulting in venous distension. These conditions can be aggravated by factors that increase venous pressure, such as prolonged standing. Given that this patient has an isolated complaint of visible "bulging veins", with no pain or warmth indicative of potential venous thrombosis, varicose veins appear to be the most likely diagnosis. However, the clinical diagnosis often needs to be confirmed with imaging. CONCLUDING: As the condition is not accompanied by potentially dangerous associated symptoms (like pain or indications of deep vein thrombosis), and given that the patient has emphasized her concern about the problem being life-threatening, it would be clinically appropriate to pursue an avenue of diagnostic confirmation to alleviate her worry, before considering treatment options. RESPONSE: Therefore, the most appropriate next step would be (D) Venous duplex ultrasonography. Venous duplex ultrasonography is a non-invasive imaging method used to visualize the anatomy of the veins, identify any abnormal flow patterns, and confirm the diagnosis of varicose veins. Other options, such as compression stockings or surgical ligation, could be considered after confirmation of the diagnosis and based on the severity of disease. Venography, a more invasive technique, isn't generally used initially. Warfarin therapy, an anticoagulant, would not be necessary as this woman doesn't exhibit signs of a venous thromboembolic event.

**AI_E:** RESTATING THE QUESTION: The query here relates to a 40-year-old woman, who after having two successful pregnancies, has been experiencing increased visibility of the veins in her legs, a condition that's been progressing for over two years. As per the physical examination, there are no abnormal signs or symptoms other than the enlarged veins, especially prominent in her lower extremities. While she is not in pain, she is not currently on any medications, and is concerned that the condition may be life-threatening. The question is, considering the options listed, what the next appropriate clinical step for this patient would be. PLANNING: To approach this question, we should consider the most likely diagnosis for this patient, which sounds like varicose veins based on symptoms of bulging veins that worsen with prolonged standing. Varicose veins are dilated, tortuous superficial veins due to chronic venous insufficiency, often associated with prolonged periods of standing, obesity, and pregnancy. We should also consider the appropriateness and effectiveness of each listed step as a treatment or further investigation for this case. CLINICAL REASONING: The patient is showing signs of apparent varicose veins, a common condition often associated with factors including prolonged standing, pregnancies, and obesity. Since she has a normal BMI and her pregnancies were uncomplicated, the most likely cause seems to be her lifestyle, perhaps involving prolonged standing. Varicose veins are typically not life-threatening but could cause uncomfortable symptoms and cosmetic disturbance. Serious complications, albeit rare, include thrombosis, bleeding, and venous ulceration. In terms of treatment, compression stockings (option A) are often a first-line treatment, as they aid in venous return, reducing the symptoms and slowing the progression of varicose veins. The other options provided, such as surgical intervention (Option B), venography (Option C), and warfarin therapy (Option E) would be more invasive, riskier, or unnecessary at this stage of her condition. Venous duplex ultrasonography (Option D), while useful in the assessment of deep venous thrombosis, is probably unnecessary in this situation as the clinical features point clearly to varicose veins and the patient has no symptoms of deep venous thrombosis (swelling, pain, redness, warmness). CONCLUDING: Therefore, in this case of varicose veins presenting with no painful symptoms, recurs consistently, worsens with prolonged standing but show no signs of more severe complications, a conservative initial treatment with compression stockings is the most appropriate next step. RESPONSE: The most appropriate next step in this scenario would be (A) Compression stockings.

**AI_D:** RESTATING THE QUESTION: The patient, a 40-year-old woman with a history of two uncomplicated pregnancies, is experiencing increasingly visible, bulging veins in her legs. These veins worsen when she stands for extended time periods but cause no pain. The patient's medical history does not reveal other significant problems, and she is not taking any medications. Her concern is that this could be a serious, potentially life-threatening condition. Her BMI is within normal range, and her vital signs are normal. Examination has revealed compressible veins in her lower extremities, more pronounced on the left, but no other anomalies. The question is asking what should be the next appropriate step in managing her condition. The choices provided are compression stockings, surgical ligation of the largest veins, venography, venous duplex ultrasonography, and warfarin therapy. PLANNING: Given her symptoms and physical findings of compressible veins in the legs, the patient likely has varicose veins. Varicose veins are dilated, tortuous, elongated superficial veins due to prolonged increased venous pressure, often in the legs. It is often of cosmetic concern and can cause symptoms like pain, swelling, heaviness, and fatigue. The condition can be exacerbated by long periods of standing, obesity, and pregnancy. However, in absence of severe symptoms, swift assessment and aggressive treatment may not be warranted. Compression stockings, which provide graduated compression of the leg muscles, can alleviate symptoms and prevent progression but they do not confirm the diagnosis. Surgical ligation or warfarin therapy are treatment options when the condition becomes severe or complicates but are not reasonable diagnostic choices or initial therapies. Venography is more invasive and currently has been replaced by duplex ultrasonography as the diagnostic tool of choice for venous disease. CLINICAL REASONING: In clinical reasoning, we must consider the option with the least invasiveness which could provide definitive diagnostic information while also accounting for the patient's lack of severe symptoms. Given this context, performing a venous duplex ultrasonography makes clinical sense. The duplex ultrasonography is non-invasive, poses minimal risk to the patient, and can confirm the diagnosis of varicose veins. It can also detect any underlying deep venous thrombosis which could present a greater risk if untreated. Since the patient does not report severe symptoms or present evidence of complications, more invasive procedures or drug therapies don't seem to be an urgent or necessary step at this point. CONCLUDING: Assuming the patient's symptoms are indeed due to varicose veins, a condition that's both prevalent and often benign, it's essential to confirm this diagnosis and rule out other severe underlying conditions. The least invasive and most informative diagnostic tool would be the venous duplex ultrasonography. This would provide a definitive diagnosis and decide the future course of treatment whether conservative, medical, or surgical. RESPONSE: The most appropriate next step would be (D) Venous duplex ultrasonography.

**Facilitator** **[Round 2]:** Summary: AI_A, AI_B, AI_C, and AI_D all agree that the most appropriate next step for the patient would be to conduct a Venous duplex ultrasonography. They all believe that the patient's condition is likely chronic venous insufficiency or varicose veins, and that a non-invasive diagnostic test is necessary to confirm this diagnosis before proceeding with treatment. They all also agree that more invasive procedures or treatments, such as surgical ligation or warfarin therapy, should be considered only after the diagnosis is confirmed and if the condition is severe or complicated. AI_E, however, believes that the most appropriate next step would be to prescribe compression stockings. AI_E also identifies the patient's condition as likely being varicose veins, but argues that since the patient's symptoms are not severe and she shows no signs of complications, a conservative initial treatment with compression stockings is more appropriate than a diagnostic test. Question: Why do AI_A, AI_B, AI_C, and AI_D believe that a diagnostic test is necessary before proceeding with treatment, while AI_E believes that a conservative treatment approach is more appropriate given the patient's symptoms and lack of complications?. Based on your preceding response, how would you answer the original question: [A 40-year-old woman, gravida 2 para 2, comes to the office because of bulging veins in her legs that have slowly become more visible since she first noticed them 2 years ago. She says they seem to worsen when she stands for long periods of time, but she reports no associated pain. She is concerned that this problem may be life-threatening. Medical history is otherwise unremarkable and she takes no medications. Both of her pregnancies resulted in uncomplicated vaginal deliveries at 40 weeks' gestation. BMI is 23 kg/m². Vital signs are normal. Physical examination discloses compressible veins in both lower extremities below the knees that are worse on the left. The remainder of the examination discloses no abnormalities. Which of the following is the most appropriate next step? (A) Compression stockings (B) Surgical ligation of the largest veins (C) Venography (D) Venous duplex ultrasonography (E) Warfarin therapy]

**AI_A:** RESTATING THE QUESTION: The case presents a 40-year-old woman with a 2-year history of increasingly visible veins in her lower extremities that worsen with standing, likely due to venous insufficiency or varicose veins. There is concern among the council regarding the most appropriate next step, whether to proceed directly to conservative treatment (compression stockings) suggested by AI_E or to conduct a diagnostic test (Venous duplex ultrasonography) favored by AI_A, AI_B, AI_C, and AI_D. PLANNING: The assumptions here are centered around the severity of the patient's condition and the best course of initial action, be it therapeutic or diagnostic. One assumption could be that, given the non-severe nature of the patient's symptoms and the absence of complications, conservative treatment should precede any diagnostic tests. An alternate assumption is that a definitive diagnosis should be established first via non-invasive diagnostic tests to guide the subsequent treatment plan. CLINICAL REASONING: Varicose veins, believed to be the patient's condition, are diagnosed primarily based on physical examination (as has been done in this case). When symptoms are severe, complications are suspected, or treatment other than compression therapy is contemplated, further testing such as duplex ultrasonography can be done. In this case, she has no severe symptoms or signs of complications. However, venturing on the side of maintaining a comprehensive approach, the precept of 'diagnosis before treatment' should be underlined. CONCLUDING: Weighing these alternate assumptions, the rationale for pursuing duplex ultrasonography would be to confirm the diagnosis, evaluate the venous system thoroughly for any disease that could influence management (e.g., deep venous thrombosis), and provide a baseline assessment against which future changes can be compared. RESPONSE: Thus, considering the above clinical reasoning, I would answer the original question with choice (D): Venous duplex ultrasonography. While a conservative treatment with compression stockings may seem appealing, confirming the diagnosis through non-invasive testing can provide a more comprehensive picture of her condition and guide further treatment if necessary.

**AI_E:** Restating the Question: This question is asking for the most optimal management plan for a 40-year-old woman presenting with progressive visibility of veins in her legs, with exacerbated symptoms upon standing, but no associated pain. Her medical history is largely unremarkable. The visible veins are a common symptom of chronic venous insufficiency or varicose veins. The available management options include compression stockings, surgical ligation of the veins, venography, venous duplex ultrasonography, or warfarin therapy. Planning: The AIs differ in whether to use non-invasive testing or intervene with conservative treatment initially. The crux lies in the assumption whether to confirm the diagnosis first or to start treatment based on the presentation alone. While most of the AIs prioritize diagnostic confirmation with Venous duplex ultrasonography, AI_E believes in commencing treatment with compression stockings. Clinical Reasoning: Chronic venous insufficiency or varicose veins are common in women who have had multiple pregnancies. The symptoms and signs presented by the patient strongly suggest this diagnosis. The AIs that suggest immediate diagnostic testing do so because of the assumption that ruling out other vascular anomalies or assessing the extent of venous insufficiency can guide subsequent management. However, considering the mild nature of symptoms currently experienced by the patient, AI_E suggests starting conservative treatment with compression stockings, as it is low-risk, has a low barrier for use and the chance of reducing symptoms. Concluding: Given the situation, both assumptions have their merits. Proceeding with immediate ultrasonography allows for the clear diagnosis of venous insufficiency and can inform additional required treatments. However, going directly to conservative therapy may be a viable option in symptomatic patients unless there’s a specific reason to confirm the diagnosis. Response: Based on the clinical history (gradual onset, visibility of the veins exacerbated by standing, and absence of associated pain) and physical exam findings (compressible veins in both lower extremities that are worse on the left with no other abnormalities), the symptoms are most likely to be varicose veins, a manifestation of chronic venous insufficiency. Given the relatively mild presentation without any alarming symptoms such as pain, bleeding, or ulceration, starting with conservative management of prescribing compression stockings (A) could be an acceptable initial step. The definitive diagnostic confirmation can follow if symptoms progress or if complications arise.

**AI_C:** The question is about the best management strategy for a 40-year old woman who is presenting with bulging veins in her legs, eluding to a probable diagnosis of chronic venous insufficiency (CVI) or varicose veins. CVI is a condition where the venous wall or valves in the leg veins are not working effectively, making it difficult for blood to return to the heart from the legs. This causes blood to pool in the veins, leading to bulging or varicosity. Severity can range from mild cosmetic issues to significant discomfort, skin changes, or even venous ulcers. Given this information, we need to assess whether diagnostic tests are required before proceeding with therapy, or whether we can directly prescribe conservative treatment. The divergence in the opinions of AI_A, B, C, D, and E seems to depend on their interpretational differences on the severity of the patient's condition. AI_A through D suggest that accurately diagnosing the condition is critical to determine the patient's treatment plan. This side of the argument assumes the patient's condition might be more severe than it initially appears. They advocate for the use of Venous duplex ultrasonography, a non-invasive technique, to confirm the diagnosis and locate the affected veins. This approach screens for complications such as deep vein thrombosis, assesses insufficiency of the valves, and maps the venous system, an important step if surgery becomes necessary. On the other side of the argument, AI_E advises a more conservative treatment strategy utilizing Compression stockings and assumes the patient's condition is not severe, given the lack of reported complications or severe symptoms. Compression stockings alleviate the symptoms of CVI and prevent the condition from getting worse without the need for invasive testing. Using a clinical reasoning approach, we determine that the gentle approach of AI_E can be taken as the initial step. However, if the patient's condition doesn't improve or worsens, we may consider implementing the approach advocated by AI_A-D, which is to perform a Venous duplex ultrasonography. This diagnostic test would help to confirm the diagnosis, rule out any potential complications, and inform any further necessary treatments. In conclusion, the preference for compression stockings (option A) as an initial treatment plan would be the most appropriate next step. This strategy would help to alleviate the symptoms for the patient while being minimally invasive, allowing for further diagnostic tests if the disease severity warrants. Therefore, I would recommend the response (A) Compression stockings.

**AI_D:** RESTATING THE QUESTION: This clinical scenario describes a 40-year-old woman who has presented with slowly progressive visible bulging veins in her legs, worse on standing but without associated pain. She has no significant medical history, takes no medications, and her pregnancies were uncomplicated. Her physical examination revealed dilated, compressible veins in both lower limbs, more pronounced on the left, with no other abnormalities. The question asks what should be done next in terms of her management. PLANNING: The clinical presentation suggests incompetence of the valves in the lower limb superficial venous system, a condition often referred to as varicose veins or chronic venous insufficiency. The long-standing nature of her symptoms, the worsening with standing (due to increased hydrostatic pressure), and the physical finding of compressible veins all point to this diagnosis. An alternate assumption might be deep venous thrombosis (DVT), but this is less likely given the lack of pain and the gradual, bilateral presentation. CLINICAL REASONING: The next step would usually be to confirm the diagnosis and assess the extent of venous incompetence. In this case, there are several options available: Venography, venous duplex ultrasonography, prescription of compression stockings, surgical ligation of the largest veins, or initiating anticoagulant therapy like warfarin. However, a thought-to-be benign condition such as varicose veins may not justify invasive procedures like venography or ligation, especially without any associated pain or complications. It seems premature and unnecessary to prescribe warfarin at this point because there is no indication of DVT or any other conditions that would necessitate anticoagulation. On the other hand, conservative management with compression stockings could certainly help alleviate her symptoms; however, confirming the diagnosis seems a wiser approach. CONCLUDING: In considering the different options, the safest and least invasive way to confirm the suspected diagnosis of varicose veins while assessing the extent of the condition is through a venous duplex ultrasonography. This technique uses ultrasound to visualize the structure and flow of blood in the veins, thereby evaluating both the anatomical and functional aspects of the venous system. It can help confirm the presence and extent of superficial venous incompetence, and even rule out the presence of a DVT. RESPONSE: Given this information, the most appropriate next step in managing this patient should be (D) Venous duplex ultrasonography. It is a non-invasive investigative tool that can confirm the diagnosis, scope the extent of the venous disease, and help guide future management decisions, all while causing minimal discomfort to the patient. The use of compression stockings (A) could certainly be part of the subsequent conservative management strategy following confirmation of the diagnosis, and invasive procedures or anticoagulant therapy should be reserved for more severe or complicated scenarios.

**AI_B:** RESTATING THE QUESTION: The query is about a 40-year-old woman who has been experiencing progressively more visible bulging veins in her legs for the past 2 years. They seem to worsen with extended periods of standing, but there are no accompanying symptoms of pain. She has reservations about this being a threat to her life. Her medical history doesn't identify any alarming potential causes, and she isn't currently on any medication. She has had two successful pregnancies, both resulting in uncomplicated vaginal deliveries at 40 weeks. She has a normal BMI, and her vital signs are in order. There are no other notable findings during her physical examination. The question is about the most appropriate next step for this patient, considering her history and presenting symptoms. PLANNING: This patient's presentation suggests conditions such as chronic venous insufficiency (CVI) or potentially varicose veins. CVI is a condition where there is long-term poor blood flow in the veins, often leading to changes in the skin, inflammation, and ulceration. Varicose veins are dilated, tortuous, and elongated veins, usually found on the legs. The leading causes of CVI include deep vein thrombosis and varicose veins. We need to confirm the diagnosis before deciding on the appropriate treatment plan. Clinically, initial conservative management with compression stockings could alleviate symptoms to an extent. This therapy can decrease venous pressure, increase venous blood flow velocity, and reduce venous blood reflux. However, if the diagnosis is confirmed as CVI, a duplex ultrasound evaluation would be the recommended tool for assessing purposes. This procedure could help delineate the pathologic process. CLINICAL REASONING: The varying opinion among the council members can be attributed to the patient's current asymptomatic condition. Those who prefer conservative management prioritize the patient's asymptomatic presentation and consider investigation excessive at the current stage if there is no sign of severe complications. On the other hand, the council members leaning towards duplex ultrasonography consider the worsening bulging veins as an indication for further investigation to rule out serious underlying conditions causing the bulging veins. The patient's quality of life could be compromised if the condition worsens, therefore a detailed investigation may prevent future severe pathologies. CONCLUDING: Given the progressive nature of the veins' bulge, coupled with its worsening with long standing periods, it could be seen as a probable sign of progression in pathology, albeit she has no pain symptoms currently. This patient's asymptomatic status doesn't necessarily indicate a lack of underlying problems, and considering the potential impact on her quality of life, immediate evaluation should be the priority. RESPONSE: Accordingly, I would propose that the most appropriate next step in this case should be (D) Venous duplex ultrasonography for better understanding and confirmation of the pathology in question. This approach would assist in both ruling out severe underlying causes and planning subsequent therapy.

**Facilitator** **[Round 3]:** Summary: AI_A, AI_B, and AI_D all agree that the most appropriate next step for the patient is to conduct a Venous duplex ultrasonography. They argue that this non-invasive diagnostic test would confirm the diagnosis, evaluate the venous system thoroughly for any disease that could influence management (e.g., deep venous thrombosis), and provide a baseline assessment against which future changes can be compared. They believe that confirming the diagnosis through non-invasive testing can provide a more comprehensive picture of her condition and guide further treatment if necessary. AI_C, on the other hand, recommends starting with conservative treatment using compression stockings. AI_C believes that the patient's condition is not severe, given the lack of reported complications or severe symptoms. Compression stockings alleviate the symptoms of CVI and prevent the condition from getting worse without the need for invasive testing. If the patient's condition doesn't improve or worsens, AI_C suggests considering the approach advocated by AI_A-D, which is to perform a Venous duplex ultrasonography. AI_E also recommends starting with conservative treatment using compression stockings. AI_E believes that given the relatively mild presentation without any alarming symptoms such as pain, bleeding, or ulceration, starting with conservative management of prescribing compression stockings could be an acceptable initial step. The definitive diagnostic confirmation can follow if symptoms progress or if complications arise. Question to Clarify Disagreements: Given the patient's current asymptomatic condition and the non-severe nature of her symptoms, is it necessary to conduct a Venous duplex ultrasonography before starting conservative treatment? Or can we start with conservative treatment and conduct the Venous duplex ultrasonography only if symptoms progress or if complications arise?. Based on your preceding response, how would you answer the original question: [A 40-year-old woman, gravida 2 para 2, comes to the office because of bulging veins in her legs that have slowly become more visible since she first noticed them 2 years ago. She says they seem to worsen when she stands for long periods of time, but she reports no associated pain. She is concerned that this problem may be life-threatening. Medical history is otherwise unremarkable and she takes no medications. Both of her pregnancies resulted in uncomplicated vaginal deliveries at 40 weeks' gestation. BMI is 23 kg/m². Vital signs are normal. Physical examination discloses compressible veins in both lower extremities below the knees that are worse on the left. The remainder of the examination discloses no abnormalities. Which of the following is the most appropriate next step? (A) Compression stockings (B) Surgical ligation of the largest veins (C) Venography (D) Venous duplex ultrasonography (E) Warfarin therapy]

**AI_A:** RESTATING THE QUESTION: The question pertains to an asymptomatic 40-year-old woman with visible, bulging veins in her legs that are exacerbated by prolonged standing. She has had two previous uncomplicated pregnancies and has no other significant medical history. Her physical examination reveals distensible veins, specifically apparent in both her lower legs but worse on the left, without other noticeable abnormalities. The goal is to decide the most appropriate next step, which can range from recommending compression stockings to conducting venous duplex ultrasonography. PLANNING: From the provided information, it is reasonable to hypothesize that this patient likely has varicose veins, a common condition associated with venous insufficiency, where risk factors include age, female sex, obesity, pregnancy, and prolonged standing. An alternate assumption would be that she might have deep vein thrombosis (DVT) as large visible veins and no pain might suggest. However, the fact that she is asymptomatic apart from visibility and worsening of the veins when standing counters this assumption. CLINICAL REASONING: Given the relatively mild presentation of the patient's condition and the lack of more severe symptoms such as pain, or higher risk factors such as obesity or a history of immobilization, the focus should be on conservative initial intervention rather than invasive diagnostic steps. Compression stockings are an appropriate first-line treatment for varicose veins and can significantly alleviate symptoms and prevent the condition from getting worse. They also have the advantage of being non-invasive. CONCLUDING: On the other hand, venous duplex ultrasonography is an excellent diagnostic test for better assessing the venous anatomy and identifying possible pathophysiology. It provides crucial objective information that aids in subsequent potential surgical interventions if they become necessary. However, given this patient's relatively benign presentation, initiating with the more patient-friendly and less costly option of compression stockings might be the most appropriate next step. RESPONSE: Therefore, (A) Compression stockings are likely the best initial action in managing this patient's condition (visible varicose veins without other symptoms). If there is no improvement or if symptoms progress, (D) Venous duplex ultrasonography should be considered to assess for additional venous disease or complications.

**AI_B:** The question is asking for the most appropriate initial management for a woman experiencing what seems to be chronic venous insufficiency (CVI), which is characterized by venous hypertension usually due to venous valve dysfunction. The proposed options include use of compression stockings, surgical ligation of the largest veins, venography, venous duplex ultrasonography, and warfarin therapy. In understanding the appropriate next step, it is necessary to consider the urgency and potential severity of the patient's symptoms, as well as the potential benefits and risks associated with each option. While some might argue that a diagnostic testing like Venous duplex ultrasonography should be the next step to confirm the diagnosis and thoroughly evaluate the venous system for any existing disease, others might posit that based on the symptoms presented i.e., bulging veins that worsen with prolonged standing and no associated pain, it's a mild case of CVI. With this in mind, a conservative approach of using compression stockings to alleviate symptoms could be initiated. The assumption to consider from a clinical perspective is whether the conservative approach would yield adequate results based on the information provided. If we question this assumption and generate an alternate one, it could be that a confirmatory diagnostic test would provide a more detailed understanding of the patient's condition and a stronger base for future treatment options. By evaluating these alternate assumptions, it becomes clear that the choice between immediate diagnostic testing and initiating conservative management stems from how severe we interpret the patient's condition. As the patient's symptoms suggest a relatively mild condition, it might be reasoned that conservative treatment is an appropriate first step. Moreover, as we move towards a conclusion, we consider the step-by-step explanation of our chosen path. If we start with compression stockings (A), this might provide immediate relief to the patient while not subjecting her to unnecessary tests. This approach could also be followed with monitoring to observe whether her symptoms improve or deteriorate and her response to the treatment. In the case where symptoms do not improve or worsen, a diagnostic test like the venous duplex ultrasonography could be considered, providing a more aggressive path. Thus, in response to the scenario, given the patient's relatively mild symptoms and lack of associated complications, a reasonable initial approach would be to start with Compression stockings (A). This provides a less invasive first step that can alleviate her symptoms while also offering an appropriate stepping stone to more intense interventions if her symptoms worsen. This conservative approach can be monitored and adjusted as necessary based on the patient's response to the treatment, ultimately providing a balance between immediate symptom relief and long-term management of her condition.

**AI_C:** The question is regarding the most suitable course of action for a 40-year-old, previously gravida 2 para 2 woman, who presents with non-painful, bulging veins in both her lower extremities. She has noticed these veins becoming more prominent over the last two years, especially when on her feet for extended periods. Despite her worry that the condition may be a serious health threat, her medical history does not reveal any abnormalities or complications, and she is not on any medication. The clinical presentation suggests Chronic Venous Insufficiency (CVI), a condition commonly linked to leg vein dysfunction due to pregnancy-induced venous distension and destruction of venous valves, which can sometimes take years to manifest symptoms. The question to ponder here is whether to suggest a diagnostic examination first before deciding the treatment plan. Should we rely on the clinical presentation and start with a conservative treatment option, or should we first conclusively diagnose the condition and then decide on treatment? When we consider the clinical reasoning, it becomes clear that her symptoms—visible, bulging veins that exacerbate on standing and are compressible on physical examination—are classic signs of CVI. Given her age, previous pregnancies, and lack of serious associated symptoms (pain, ulceration, bleeding), her case appears to be of a mild CVI. It is also worth noting that the risk of undergoing an invasive diagnostic test (venography) or an unnecessary potential invasive treatment (surgical ligation of the largest veins) may outweigh the benefits for her at this stage. Warfarin therapy is a treatment for Deep Vein Thrombosis and does not appear to be indicated in this clinical scenario. Thus, a conservative initial approach seems to be a reasonable option. We can start with compression stockings, a non-invasive, simple, and cost-effective way to mitigate symptoms associated with CVI. The purpose of these stockings is to provide graded pressure that assists in directing the blood flow from the legs back to the heart, thereby reducing discomfort and preventing progression of the condition. But, if her symptoms fail to improve or worsen, an evaluation with Venous duplex ultrasonography might be necessary. It is a non-invasive test that will not only help to confirm the diagnosis but also assess the venous system for any comorbidities that might influence treatment management, such as detecting the presence of deep venous thrombosis. In conclusion, given the patient's minimal symptoms, lack of complications, and the non-threatening severity of her current condition, as well as considering the ability to diagnose and potentially treat CVI initially with a conservative approach, option (A) Compression stockings seems to be the most appropriate next step. If her condition worsens or fails to improve with this approach, a Venous duplex ultrasonography may subsequently be required.

**AI_D:** Restating the question: This question asks us to determine the best initial approach for a 40-year-old woman, who is manifesting visible varicosities in both her lower extremities with more predominance on the left side. More importantly, these varicosities have slowly become more visible over the past 2 years, and seems to worsen when she's standing for long. Notably, they are painless, and she has no other complaints. Her medical history is clear, and she has had two pregnancies with normal deliveries. She's currently not on any medications, with a body mass index (BMI) within normal range. Planning: A significant thing to note here is that varicose veins are usually benign and commonly occur in the legs, particularly in women who have borne children. The question stipulates whether conservative treatment should be initiated first or if a diagnostic test should be done prior. One could then project two possible assumptions: (1) conservative management (compression stockings) would provide immediate relief and control of the mild symptoms, or (2) performing a Venous duplex ultrasonography will permit a more precise diagnosis and any other underlying abnormalities. Clinical reasoning: Given the restatement and assumptions, varicose veins result from incompetent venous valves, leading to blood pooling and venous dilatation. In this case, the patient's history of pregnancies could have predisposed her to acquiring these varicose veins. Hence, starting conservative treatment might give immediate relief and control of symptoms. This assumption is grounded in the fact that compression stockings are known to alleviate symptoms caused by venous stasis and promote better venous flow by applying graduated pressure on the legs. But then, varicose veins can also coincide with other venous diseases, such as deep vein thrombosis (DVT). One should then opt for venous duplex ultrasonography as another assumption because this non-invasive procedure will be the most efficient approach to confirm the diagnosis of varicose veins. In addition to that, it will also monitor for any co-existing diseases like DVT that could affect the management of the patient. Concluding: In essence, one could approach the clinical situation either through conservative management or through a diagnostic approach. However, visible bulging veins that worsen on prolonged standing, although painless, needs to be critically evaluated. The duplex ultrasonography can recapitulate the scenario by confirming the presence of varicosity and any co-existing venous disease. Further, the duplex ultrasonography can provide a baseline assessment to which any future changes could be compared. Response: Hence, the most suitable next step would be (D) Venous duplex ultrasonography. This approach makes sure that we're not only treating the visual symptoms the patient is exhibiting but also checking for possible underlying conditions that might affect her overall treatment and management. Furthermore, this gives an accurate description of the situation of her venous health so that more personalized care can be provided if needed.

**AI_E:** RESTATING THE QUESTION: This patient, who is [A 40-year-old woman, gravida 2 para 2, with no significant medical history and no current medications], is concerned about bulging veins in her legs that have become progressively visible over the last 2 years and worsen when she stands for long periods. furthermore, she's alarmed that this problem could be life-threatening. She is physically healthy with a normal BMI and vital signs. On examination, compressible veins were noticed in both lower extremities below the knees, particularly on the left. There are no other abnormalities. We are asked to determine the most appropriate next step given these findings. PLANNING: The likely medical condition here is chronic venous insufficiency (CVI), a disorder where veins cannot pump enough blood back to the heart. This issue is usually due to damaged valves in the veins. It often affects legs, where gravity can cause blood to pool. This woman's symptoms – lack of pain, worsened venous visibility on standing, and compressible veins in both lower extremities – align with such a diagnoses. Some may argue for options like Venous duplex ultrasonography or Venography to help confirm the diagnosis and look into the venous system more intensively for any anomalies that could affect management. Others may advocate for a more conservative approach and suggest compression stockings without an immediate necessity for diagnostic testing, given the mild presentation without pain, bleeding, or ulceration. CLINICAL REASONING: Chronic venous insufficiency is often diagnosed based on a person's symptoms and a physical examination. The compressible veins in both lower extremities of the patient are a visible sign of CVI, and her symptom of them worsening when she stands for long periods is also a common symptom. The absence of pain, bleeding, or ulcerations suggests a relatively mild progression so far. Venous duplex ultrasonography or Venography could be very informative, as it could help confirm the diagnosis and provide a more comprehensive picture of her condition to guide treatment. But it's good to consider the invasiveness, cost, and potential unnecessary implications for the patient, especially given her relatively mild presentation and absence of associated symptoms. On the other hand, starting with conservative treatment using compression stockings could help reduce symptoms, potentially decrease disease progression, and avoid the need for more invasive procedures. This management strategy would prioritize the patient's current uncomplicated condition and limit potential risks associated with unnecessary procedures. CONCLUDING: Given the mild presentation of the woman's condition, her symptom progression over 2 years, and the absence of severe symptoms that could indicate complications, it seems more appropriate to start with conservative management in the form of compression stockings. This should alleviate her symptoms while monitoring her progress over time. If her condition worsens or new symptoms arise, then we can consider conducting a venous duplex ultrasonography to obtain a more complete picture of her venous system to guide further management. RESPONSE: Therefore, based on the woman's symptoms, physical examination findings, and course of symptom progression, the most appropriate next step would be to start with conservative treatment. This conservative treatment would be (A) Compression stockings, which could help her manage the condition without introducing the need for the more invasive procedures at this time.

**Facilitator** **[Round 4]:** Summary: AI_A, AI_B, AI_C, and AI_E all agree that the patient's symptoms suggest a mild case of Chronic Venous Insufficiency (CVI) and recommend starting with a conservative approach using compression stockings. They argue that this non-invasive treatment can alleviate symptoms and prevent the condition from worsening. If the patient's condition does not improve or worsens, they suggest considering venous duplex ultrasonography to assess for additional venous disease or complications. AI_D, however, recommends starting with venous duplex ultrasonography. They argue that this diagnostic test will provide a more precise diagnosis and identify any other underlying abnormalities. They believe that this approach will ensure that the patient's treatment and management are based on a comprehensive understanding of her venous health. Key Differences: The key difference between the perspectives lies in the perceived severity of the patient's condition and the appropriate initial response. AI_A, AI_B, AI_C, and AI_E believe that the patient's symptoms suggest a mild case of CVI and that a conservative approach using compression stockings is the most appropriate initial response. They argue that this approach can alleviate symptoms, prevent the condition from worsening, and avoid unnecessary invasive procedures. AI_D, on the other hand, believes that the patient's condition warrants a more thorough initial investigation. They argue that venous duplex ultrasonography will provide a more precise diagnosis and identify any other underlying abnormalities, ensuring that the patient's treatment and management are based on a comprehensive understanding of her venous health. Question for Council Members: Given the patient's symptoms and the potential risks and benefits of both approaches, how would you weigh the importance of immediate symptom relief through conservative treatment against the potential for a more comprehensive understanding of the patient's condition through venous duplex ultrasonography?. Based on your preceding response, how would you answer the original question: [A 40-year-old woman, gravida 2 para 2, comes to the office because of bulging veins in her legs that have slowly become more visible since she first noticed them 2 years ago. She says they seem to worsen when she stands for long periods of time, but she reports no associated pain. She is concerned that this problem may be life-threatening. Medical history is otherwise unremarkable and she takes no medications. Both of her pregnancies resulted in uncomplicated vaginal deliveries at 40 weeks' gestation. BMI is 23 kg/m². Vital signs are normal. Physical examination discloses compressible veins in both lower extremities below the knees that are worse on the left. The remainder of the examination discloses no abnormalities. Which of the following is the most appropriate next step? (A) Compression stockings (B) Surgical ligation of the largest veins (C) Venography (D) Venous duplex ultrasonography (E) Warfarin therapy]

**AI_C:** The woman in the scenario is presenting with bulging veins in her legs, particularly worse when standing, which is a manifestation of chronic venous insufficiency (CVI). This condition is the result of impaired venous return, leading to chronic increased venous pressure and ultimately impacting the venous valves' function. It often presents with various symptoms such as leg swelling, pain, restlessness, skin changes, and in severe cases, venous ulcers. However, the woman does not complain of any pain or discomfort, indicating that her case might be in the early stages of this condition. Two considerations arise here: firstly, the severity of CVI, and secondly, the patient's concern about how life-threatening this condition could be. Although the lack of pain and other severe symptoms may indicate a mild case of CVI, her fear and anxiety about the situation certainly are significant elements influencing her quality of life. The two suggested actions seem to revolve around these issues: compression stockings as immediate palliative care and venous duplex ultrasonography for a thorough investigation and confirmation of CVI. Compression stockings are a non-invasive method, effective in improving the symptoms of CVI by increasing venous return, reducing venous dilation, and thus lessening the long-term sequelae. They also provide immediate relief and reassurance to the patient without being invasive or carrying risks associated with surgical or pharmacological intervention. Venous duplex ultrasonography, on the other hand, would be a means to confirm the diagnosis and the extent of her venous abnormality, giving a clearer picture of her underlying condition and thus would lower her anxiety by providing concrete information. Keeping these two principles in mind – relieving the patient's immediate concerns (both physical and emotional) and establishing a sound diagnosis – I would lean towards starting with compression stockings (A) as the initial step. This method would address her current distress, provide possible symptom relief, and not impose any immediate risks, fitting the criteria of the "First do no harm" principle in medicine. However, I would also inform the patient about the option of Venous duplex ultrasonography (D) as a next step to achieving an appropriate diagnosis in the event of no improvement or worsening of her condition. With this plan, we would be providing immediate care while keeping an open path for a more thorough investigation should it become necessary.

**AI_E:** Restating the Question: This 40-year-old woman has developed noticeable bulging veins in her legs that worsen with standing and have been progressing over the past two years. Given her symptom of prominent superficial veins without any associated pain or other medical conditions, one plausible diagnosis is chronic venous insufficiency (CVI). CVI is a condition characterized by impaired venous return, leading to increased venous pressure and subsequent vein distention. The question is asking us to determine the next appropriate step in management or evaluation of her condition, with options that range from a conservative approach to direct intervention and diagnostic procedures. Planning: Assumptions under consideration include that the patient has CVI and it is a mild case given her limited symptoms. This could mean that conservative measures such as compression stockings may alleviate her symptoms and prevent further progression. Alternatively, we could hypothesize that her condition warrants further investigation to understand the extent of the venous disease or to rule out other associated conditions. Clinical Reasoning: Assuming this is a case of CVI, the use of compression stockings (option A) could help improve venous return, alleviate her symptoms, and slow the progression of the disease. This conservative treatment approach often is considered valuable when CVI symptoms are mild and absent of other complicating factors, as in her case. On the other hand, considering a diagnostic procedure such as venous duplex ultrasonography (option D) for further evaluation can provide a more comprehensive understanding of the patient's venous health. It is quite useful in diagnosing the presence of venous reflux or obstructions and can help give an anatomical overview of her venous system. However, this may not be required immediately given her benign presentation. Concluding: Connecting these thoughts, a mild case of suspected chronic venous disease can initially be managed conservatively with compression stockings. This treatment option is easy to implement, non-invasive, and can provide symptom relief. Moreover, observing the patient's response to this intervention can give more insight into her vascular health. However, if there's no improvement or the condition worsens, a duplex ultrasound may then be necessary. Response: After considering all factors, the most appropriate initial step would be to start this patient on compression stockings (option A) given her symptom presentation. This provisional approach can be beneficial in alleviating her symptoms and decelerating any further disease progression. This way, we can avoid unnecessary interventions and also give us some time to further evaluate her condition. Duplex ultrasonography could be a next step should her condition not improve or worsen, or if the conservative approach does not work.

**AI_B:** Restating the Question: The question is about a 40-year-old woman who is showing signs of bulging veins in her legs that intensify the longer she stands. Despite their increased visibility over the past two years, they do not cause pain. She does not have any other significant medical history. Because these veins become more prominent when she's standing and because they are compressible, my inclination is towards a diagnosis of Chronic Venous Insufficiency (CVI). Planning: We should bear in mind that the patient is concerned that these veins might pose a life-threatening situation. It's also important to remember that, other than these venous changes, her medical status is unremarkable. Her pregnancies have not had any complications and her vitals are within the normal range. Regarding the question's alternatives, compression stockings can provide symptomatic relief for less severe cases of CVI. Venous duplex ultrasonography is a diagnostic tool that can assess the patency and functionality of the venous system more precisely. Venography, while considered the gold standard for venous disease diagnosis, is more invasive and could be reserved for cases in which the condition cannot be confirmed by ultrasonography. Surgical ligation and warfarin would be too extreme based on the current scenario; without a known deep vein thrombosis (DVT), coagulation therapy would not be appropriate, and surgical intervention should be considered only for cases that are resistant to conservative management. Clinical Reasoning: Based on the data provided, it seems that the symptoms are related to a possible CVI or varicose veins - conditions that are typically benign and unlikely to be life-threatening. Standing for long periods often contributes to the discomfort and visibility of varicose veins due to blood pooling in the lower extremities, causing the veins to bulge. As there is no sign of imminent danger like severe pain, swelling, or discoloration indicative of a more serious condition like DVT, a conservative approach could be initially pursued. Concluding: In conclusion, given the current clinical scenario, conservative management such as compression stockings, lifestyle changes, or leg elevation could be a beneficial starting point to provide symptomatic relief, delay disease progression, and alleviate the patient's fears. More invasive diagnostic or treatment modalities, such as venous duplex ultrasonography or venography, could be reserved for cases where symptoms do not improve or worsen over time, or where there is a clinical suspicion for more serious venous diseases. Response: The response to the question: [A 40-year-old woman, gravida 2 para 2, comes to the office because of bulging veins in her legs... Which of the following is the most appropriate next step?] would be (A) Compression stockings.

**AI_D:** Restating the question: A woman of reproductive age has presented with a concern about noticeable veins in her legs that worsen after standing. There is no pain associated with these veins nor any abnormalities seen in her physical examination. We are tasked with determining the most appropriate next step in her care. These visible veins likely refer to varicose veins, which are abnormally dilated superficial veins often seen in legs. Planning: The assumptions being made here include the idea that these bulging veins are harmless varicose veins, that these varicose veins might be part of a larger problem such as chronic venous disease (CVD), or that immediate intervention is required on the basis of her distress. An alternative assumption that could be generated may be that while she is presenting solely with varicose veins, she could have a more severe or underlying condition that would require more extensive investigation. The assumption to clinically prioritize is the consideration of the spectrum of CVD. Clinical reasoning: Chronic venous disease is part of a spectrum that runs from spider veins and varicose veins, through chronic venous insufficiency, to chronic venous stasis ulcers. It's caused by degradation or damage to the one-way valves in the veins which prevent blood from flowing backward when the muscles aren't contracting. When these valves start becoming inefficient, blood can pool in the vein, creating the visible bulging. Severe or unmanaged CVD can lead to changes in the skin, ulcers and possibly a deep vein thrombosis (DVT). Varicose veins can be associated with a slight increase in the risk of DVT. In this case, the patient's symptoms and presentation are typical for varicose veins without indication of complications or CVD. Concluding: In terms of management, the recommended first-line treatment for varicose veins without complications such as notable edema, skin changes or ulcers, is conservative care. This includes leg elevation, exercise, and compression stockings, which can relieve symptoms and prevent progression of the disease. More invasive procedures, such as surgical ligation, are considered if conservative measures are not effective or if the patient has more severe disease. Considering these factors, further diagnostic measures would not be necessary unless the initial recommended treatments are unsuccessful or if her condition worsens. Response: Therefore, given her presentation and the lack of signs of severe disease, it would seem that the most appropriate next step is to recommend conservative treatment. This involves the use of compression stockings, making choice (A) "Compression stockings" the most suitable next step for this patient. Her concern for the life-threatening consequence can be reassured with proper explanation on the condition.

**AI_A:** Restating the Question: The patient, a 40-year-old woman, gravida 2 para 2, has come to the doctor with concerns about increasingly visible veins in her legs over the past two years. These veins bulge more when she stands for a substantial amount of time, causing her to worry about it being a life-threatening issue. However, she does not experience pain, her medical history appears normal, and she's currently not on any medications. Additionally, the pregnancies she had resulted in uncomplicated vaginal deliveries. Her BMI is within a healthy range and all her vital signs are normal as well. A physical exam shows compressible veins in both her legs below the knees, more so on the left, but there are no other notable abnormalities. We are asked to decide on the best course of action for this patient. Planning: The visible bulging veins suggest that given her pregnancies the woman likely has chronic venous insufficiency (CVI) as this is a common condition found among women who have been pregnant. CVI is characterised by pooling of blood in the veins, usually in the legs. It usually presents with symptoms such as visible varicose veins, swelling of the legs, and a sensation of heaviness. However this is an assumption assuming from symptoms alone without clinical diagnostic evidence. Considering the violent means that could be available to handle the situation, using compression stockings would be a non-invasive first step to help manage the condition. They could provide symptom relief and slow the condition's progression. If her condition worsens or doesn't improve, venous duplex ultrasonography might be suggested to ascertain additional venous disease or complications. This reasoning considers the possibilities that it could be a simple, manageable condition mission of clinical diagnosis could change the course of treatment. Clinical Reasoning: Upon initial assessment, her symptoms and presentation seem indicative of a mild case of CVI. The slow progression of visible veins over the years, the absence of pain, her reproductive history, and the physical exam findings support this conclusion. We have two primary clinical strategies to consider here: (1)a conservative, symptom-based approach with the use of compression stockings, or (2) a diagnostic-focused approach with the use of venous duplex ultrasonography which has its own risks and benefits. The conservative approach could manage her symptoms, slow progression, and avoid invasive procedures. The diagnostic-focused approach could provide a sharper diagnosis, potential additional findings, and a more targeted treatment approach. Concluding: Upon evaluation of both strategies and consequentially considering the patient's perspective as she only has concerns about the visibility of the veins and there is no pain, the focus should be on immediate symptom relief. Thus, using compression stockings would be the most appropriate initial response. Notwithstanding, if the condition does not improve or worsens, venous duplex ultrasonography should be considered to investigate for any other venous disease or complications that might have been initially overlooked. Response: Given this patient's symptoms, concern, absence of pain, and normal history, the most appropriate initial step would be the application of compression stockings (Option A). They will not only offer the patient relief but also prevent the condition from worsening. However, if the symptoms persist or worsen, further investigation would be necessary, and venous duplex ultrasonography may be considered.

**Facilitator** The consensus among the AI responses is that the 40-year-old woman's symptoms of bulging veins in her legs, which worsen when standing, suggest a likely diagnosis of Chronic Venous Insufficiency (CVI). This condition is characterized by impaired venous return, leading to increased venous pressure and subsequent vein distention. Given her symptom presentation, absence of pain, and normal medical history, the most appropriate initial step would be conservative management with compression stockings (Option A). This non-invasive method can provide immediate symptom relief, slow the progression of the disease, and alleviate the patient's concerns without imposing any immediate risks. However, if her condition does not improve or worsens, further investigation would be necessary. In such a case, venous duplex ultrasonography (Option D) may be considered to provide a more comprehensive understanding of her venous health and to diagnose the presence of venous reflux or obstructions. More invasive procedures, such as surgical ligation or venography, and pharmacological interventions like warfarin therapy, are not recommended at this stage given her benign presentation and the absence of severe disease signs.
